# Intraoral scanners in dentistry: a review of the current literature

**DOI:** 10.1186/s12903-017-0442-x

**Published:** 2017-12-12

**Authors:** Francesco Mangano, Andrea Gandolfi, Giuseppe Luongo, Silvia Logozzo

**Affiliations:** 1 Department of Medicine and Surgery, Dental School, University of Varese, Varese, Italy; 2 Private Practice, Bologna, Italy; 3 Department of Oral and Maxillofacial Surgery, Federico II University, Naples, Italy; 4 Department of Engineering, University of Perugia, Perugia, Italy; 5 V-GER srl, Department of Research and Development, Bologna, Italy

**Keywords:** Intraoral scanners, Optical impressions, Accuracy, Time efficiency, Clinical use

## Abstract

**Background:**

Intraoral scanners (IOS) are devices for capturing direct optical impressions in dentistry. The purpose of this narrative review on the use of IOS was to: (1) identify the advantages/disadvantages of using optical impressions compared to conventional impressions; (2) investigate if optical impressions are as accurate as conventional impressions; (3) evaluate the differences between the IOS currently available commercially; (4) determine the current clinical applications/limitations in the use of IOS.

**Methods:**

Electronic database searches were performed using specific keywords and MeSH terms. The searches were confined to full-text articles written in English and published in peer-reviewed journals between January 2007 and June 2017.

**Results:**

One hundred thirty-two studies were included in the present review; among them, 20 were previous literature reviews, 78 were in vivo clinical studies (6 randomized controlled/crossover trials, 31 controlled/comparative studies; 24 cohort studies/case series; 17 case reports) and 34 were in vitro comparative studies.

**Conclusions:**

Optical impressions reduce patient discomfort; IOS are time-efficient and simplify clinical procedures for the dentist, eliminating plaster models and allowing better communication with the dental technician and with patients; however, with IOS, it can be difficult to detect deep margin lines in prepared teeth and/or in case of bleeding, there is a learning curve, and there are purchasing and managing costs. The current IOS are sufficiently accurate for capturing impressions for fabricating a whole series of prosthetic restorations (inlays/onlays, copings and frameworks, single crowns and fixed partial dentures) on both natural teeth and implants; in addition, they can be used for smile design, and to fabricate posts and cores, removable partial prostheses and obturators. The literature to date does not support the use of IOS in long-span restorations with natural teeth or implants. Finally, IOS can be integrated in implant dentistry for guided surgery and in orthodontics for fabricating aligners and custom-made devices.

## Background

Intraoral scanners (IOS) are devices for capturing direct optical impressions in dentistry [1–3]. Similar to other three-dimensional (3D) scanners, they project a light source (laser, or more recently, structured light) onto the object to be scanned, in this case the dental arches, including prepared teeth and implant scanbodies (i.e. cylinders screwed on the implants, used for transferring the 3D implant position) [2, 3]. The images of the dentogingival tissues (as well as the implant scanbodies) captured by imaging sensors are processed by the scanning software, which generates point clouds [3, 4]. These point clouds are then triangulated by the same software, creating a 3D surface model (mesh) [3, 4]. The 3D surface models of the dentogingival tissues are the result of the optical impression and are the ‘virtual’ alternative to traditional plaster models [4, 5].

Although IOS are becoming widespread in clinical dental practice, only a few reviews on the use of these devices are available in the literature [5–8].

The purpose of the present narrative review was therefore to:

▪ identify the advantages and/or disadvantages of using optical impressions compared to conventional impressions;

▪ investigate if optical impressions are as accurate as conventional impressions;

▪ evaluate the differences between the IOS currently available commercially;

▪ determine the current clinical applications and limitations in the use of IOS;

taking into consideration all studies currently available in the scientific literature.

## Methods

### Study design

At present, it is difficult to conduct a complete systematic review of IOS, given the insufficient number of randomised controlled trials available on the clinical use of these devices as well as the numerous possible clinical applications and the technological elements to be considered; authors who have attempted to address this topic systematically in fact focused on specific clinical applications of IOS [6] and/or had difficulties finding sufficient randomised controlled trials to include in their systematic reviews [5, 7, 8]. For these reasons, we decided to perform a narrative review and attempt to answer a series of focused questions that may be of interest to the reader. In fact, these focused questions enable the investigation of the indications (and contraindications) for the clinical use of IOS, as well as the most important technological features of these devices, providing the reader a detailed overview of the subject.

The focused questions are:

1. *What are the advantages and disadvantages of optical impressions with respect to conventional impressions?*
2. *Are optical impressions as accurate as conventional impressions?*
3. *What are the differences between the optical impression systems available commercially?*
4. *To date, what are the clinical applications of IOS?*

This narrative review was prepared and written based on the indications that emerged during the State of the Art of Digital Technologies in Daily Dental Practice Consensus Conference of the Digital Dentistry Society (DDS) held in Milan in September 2016.

### Search strategy

The protocol of this narrative review recognised that in vivo studies are the most appropriate to address a focused question that embraces the clinical effectiveness of IOS. However, as IOS have been recently introduced commercially, and as it is not possible to mathematically evaluate the accuracy of IOS in vivo, both in vivo and in vitro studies were included in the hierarchy of evidence for this review. Among the in vivo studies, both experimental (randomized controlled/crossover trials) and observational (controlled/comparative studies, prospective/retrospective cohort studies, case series and case reports) clinical studies were eligible for this review. Electronic database searches of MEDLINE, Embase and Scopus were performed using keywords and MeSH terms based on a search strategy used for searching MEDLINE (via PubMed): (((intraoral scanners OR digital impressions OR optical impressions OR intraoral scanning systems)) AND ((accuracy OR trueness OR precision OR time efficiency OR reliability))). The searches were confined to full-text articles written in English and published in peer-reviewed journals between January 2007 and June 2017. Titles and abstracts were screened and then full texts of all potentially relevant publications were obtained and reviewed independently in duplicate by F. Mangano and S. Logozzo, who also performed the data extraction. The investigators recorded the study title, authors, year of publication, journal in which the research was published and study design and type (in vitro or in vivo research). For in vitro studies, the investigators recorded subject area, materials, number of samples, outcomes, statistical findings and conclusions. For in vivo clinical studies, the investigators recorded subject area, randomisation and/or blinding where present, number of patients treated, controls (if present), treatment phases, follow-up, results, statistical findings and conclusions. Finally, the two independent investigators reached consensus for the inclusion of researches in this review.

## Results

### Search results and included studies

In total, 132 studies were included in the present literature review. These articles were published over a 10-year period, i.e. between January 2007 and July 2017, and demonstrated considerable variation with respect to study type, study design and results. Among these studies, 20 were previous literature reviews, 78 were in vivo clinical studies (6 randomized controlled/crossover trials, 31 controlled/comparative studies; 24 cohort studies/case series; 17 case reports) and 34 were in vitro comparative studies.

### Focused questions

#### 1. What are the advantages and disadvantages of optical impressions with respect to conventional impressions?

The advantages and disadvantages of optical impressions with respect to conventional physical impressions (i.e. impressions made with trays and materials) are presented below and summarised in Table 1.

**Table 1:** Advantages and disadvantages of optical impressions according to the current literature

Advantages	Disadvantages
Less patient discomfort [2, 4, 6, 7, 9–18]	Difficulty detecting deep marginal lines of prepared teeth [2–5, 26, 29–33]
Time-efficient [6, 13, 15, 16, 18–24]	Learning curve [29–34]
Simplified clinical procedures [2, 6, 20–24, 26–30]	Purchasing and managing costs [2–5]
No more plaster casts [2, 4, 6, 20, 22, 23, 25–30]	
Better communication with the dental technician [2, 4, 6, 23, 25–30]	
Better communication with patients [2, 4, 6, 20–24, 26–32]	

##### Less patient discomfort

The ability to directly capture all dental arch information of the patient, and consequently their 3D models, without using conventional physical impressions, is one of the advantages of optical impressions [1, 4, 7, 8]. In fact, the conventional physical impressions can cause momentary discomfort for the patient due to the inconvenience and hardship stemming from the materials positioned on impression trays (whether generic or individualised) [1, 4, 7–11]. Some patients (e.g. patients with strong gag reflex, or children) appear not to tolerate the classic procedure [2, 3, 9–11]. For such patients, replacing conventional impression materials with light is an advantage; optical impression is therefore appreciated [9–12]. Optical impression decreases patient discomfort significantly when compared to traditional physical impression [13–19]. In fact, it eliminates the need for materials and impression trays, which are often unwelcome to the patient [9–11, 13–19]. Patients tend to prefer optical impressions rather than conventional impressions, as reported by the literature [12–19].

##### Time efficiency

Several studies have shown that optical impressions are time-efficient, as they enable reduction of the working times (and therefore costs) when compared to conventional impressions [6, 13, 15, 16, 18–24]. Despite the recent technological advancements in IOS, with the latest devices introduced in the market enabling the capture of a full-arch scan in less than 3 min, it does not appear that the major differences in time efficiency stem from the act of making an impression itself (a full-arch scan may take 3–5 min, similar to that required for conventional impressions), but rather from the time saved afterwards, during all subsequent steps [6, 16, 20, 25]. In fact, with optical impressions, there is no need to pour stone casts and obtain physical plaster models [2, 5–7, 13, 15, 16, 19–24]; it is possible to e-mail the 3D virtual models (proprietary or. STL files) of the patient directly to the dental laboratory without the need to deliver anything via courier or regular mail [4–6, 8, 13, 15, 16, 18–24]. This enables the saving of a considerable amount of time and money during the working year [4–6, 8, 13, 15, 16, 18–24]. For dental clinics equipped to design and manufacture of chair-side prosthetic restorations, the files captured during optical impressions may be imported into computer-assisted design (CAD) software; once the restoration design is completed, the files can be transferred to computer-assisted manufacturing (CAM) software and put into the milling machine. The restorations (in different materials) thus obtained will be characterised and ready for clinical application [4, 6, 14, 16, 19–22].

##### Simplified procedures for the clinician

Another benefit conferred by the use of optical impression is clinical [2, 6, 20–24, 26–30]. In fact, when the learning curve has been completed [31, 32], the use of IOS may confer further clinical advantages, simplifying impression-making in complex cases, for example in the presence of multiple implants or severe undercuts that may render the detection of a conventional impression difficult and insidious [2, 6, 20–30]. Moreover, if the clinician is not satisfied with some of the details of the recorded optical impression, they may delete them and recapture the impression without having to repeat the entire procedure; this aspect is time-saving [2, 6, 20, 22, 23, 25–32].

##### No more plaster casts

For the clinician, optical impression allows the skipping of an otherwise unavoidable step (the conventional impression is based on the detection of physical impressions and subsequent casting of gypsum models) with a time-saving effect [2, 4, 6, 20, 22, 23, 25–30]. The elimination of conventional impression materials translates into direct savings for the clinician, with reduced consumables costs [2, 4, 6, 20, 22, 23, 25–32].

##### Better communication with the dental technician

With IOS, the clinician and the dental technician can assess the quality of the impression in real-time [2, 4, 6, 20, 22, 23, 25–30]. In fact, immediately after the scan has been performed, the dentist can e-mail it to the laboratory, and the technician can check it accurately [2, 22–24, 26–30]. If the dental technician is not convinced of the quality of the received optical impression, he/she can immediately request that the clinician make another one without any loss of time and without having to call the patient for a second appointment [2, 4, 6, 23, 25–30]. This aspect simplifies and strengthens communication between the dentist and dental technician [2, 4, 6, 23, 25–30].

##### Better communication with patients

Optical impression is a powerful tool for patient communication and marketing [2, 4, 6, 20–24, 26–32]. In fact, with optical impressions, patients feel more involved in their treatment and it is possible to establish more effective communication with them; this emotional involvement may have a positive impact on the overall treatment, for example, by improving patient compliance to oral hygiene. In addition, patients are interested in the technology and mention it to their acquaintances and friends, raising their consideration of dental centres equipped with these modern technologies. Indirectly, IOS has become a very powerful advertising and marketing tool [2, 4, 6, 20–24, 26–30].

##### Learning curve

There is a learning curve for adopting IOS in the dental clinic, and this aspect must be considered with attention [29–34]. Subjects with a greater affinity for the world of technology and computers (e.g. young dentists) will find it very easy to adopt IOS in their practice. Older clinicians with less experience and passion for technological innovations could find using the devices and related software more complex for [29–34]. Lastly, it should be kept in mind that it is still unclear whether one scanning strategy is better than the other, as manufacturers provide little information about their scanning strategies. This is an aspect that will certainly be researched in-depth in the coming years, as it is possible that different machines, using different scanning strategies, would produce different results.

##### Difficulty detecting deep margin lines of prepared teeth

One of the most frequent problems encountered with IOS and with optical impressions is difficulty in detecting deep marginal lines on prepared teeth or in the case of bleeding [2–5, 26, 29–32]. In some cases, in fact, and especially in aesthetic areas where it is important for the clinician to place the prosthetic margins subgingivally, it may be more difficult for the light to correctly detect the entire finishing line [2–5, 26, 29–32]. In fact, unlike the conventional impression materials, light cannot physically detach the gum and therefore cannot register ‘non-visible’ areas. Similar problems can also occur in the event of bleeding, as blood may obscure the prosthetic margins [2, 26, 29–32]. Despite this, with the proper attention and speed (the gingival sulcus tends to close immediately after the retraction cord is removed) and the appropriate strategies for highlighting the preparation line (insertion of a single or double retraction cord), and avoiding bleeding (excellent oral hygiene and provisionals with correct emergency profile), it is possible for the clinician to detect a good optical impression even in difficult contexts [1, 2, 5]. Recently, some authors have suggested combining strategies, i.e. partly using conventional impression materials [33]. Beyond that, a good optical impression is the result of many factors, namely the quality of prosthetic preparation, the patient’s compliance with oral hygiene, and the goodness of the provisional restorations; as with conventional impressions, healthy soft tissues are essential for a good optical impression [33, 34]. These considerations are all valid for natural teeth, but not for dental implants, where the use of scanbodies (accurately coupled with CAD-related calculations) solves any problem.

##### Purchasing and managing costs

Depending on the model, the cost of purchasing an IOS may be between 15.000 and 35.000 euros. Over the last few years, manufacturers have released many new models on the market, and the growth in supply should be accompanied by a reduction in purchase costs [1–5]. Regardless, the purchase cost of a high-end, last-generation IOS should be cushioned over the year by integrating the device into the clinical workflow across the various dental disciplines (prosthodontics, orthodontics, implant surgery) [1–5]. One important aspect to consider is additional managing costs related to upgrades of the reconstruction software. Different manufacturing companies have different policies in this regard, and it is important for the clinician to be fully informed of the annual management costs and fees, where present, before purchasing an IOS [2–5]. Finally, in the case of ‘closed’ systems, or with IOS that output only proprietary file formats, an annual or monthly fee may be required to ‘unlock’ the files and render them usable by any CAD software or any laboratory. Once again, the clinician should be properly informed about these additional managing costs.

#### 2. Are optical impressions as accurate as conventional impressions?

The main feature an IOS should have is accuracy: a scanner should be able to detect an accurate impression [1–8]. In metrics and engineering, accuracy is defined as the ‘closeness of agreement between a measured quantity value and a true quantity value of a measurand’ (JCGM 200:2012, ISO 5725–1, 1994). Ultimately, accuracy is the sum of trueness and precision [4–8]. Trueness, usually expressed in terms of bias, is the ‘closeness of agreement between the expectation of a test result or a measurement result and a true value’ [4–8]. Precision is defined as the ‘closeness of agreement between indications or measured quantity values obtained by replicate measurements on the same objects under specified conditions’ [4–8]. Ideally, an IOS should have high trueness (it should be able to match reality as closely as possible). An IOS should therefore be as true as possible, that is, be able to detect any impression detail and permit the establishment of a virtual 3D model as similar as possible to the actual model, and that little or nothing deviates from reality. The only means of calculating the trueness of an IOS is to overlap its scans with a reference scan obtained with a powerful industrial machine (industrial optical scanner, articulated arm, coordinate measuring machine) [4–8]. After the overlapping of these images/models, powerful reverse-engineering software can be used to generate colorimetric maps displaying the distances/differences between the surfaces of the IOS and the reference model at micrometric level [4]. Precision can be calculated more easily, simply by overlapping different scans/models taken with the same IOS at different times and again evaluating the distances/differences at micrometric level. Technically, an IOS could have high trueness but low precision, or vice versa. In both cases, the optical impressions would be unsatisfactory: this would negatively affect the entire prosthetic workflow, where reducing the marginal gap is the prosthodontist’s major task. Trueness and precision mainly depend on the scanner acquisition/processing software, which performs the most difficult task: ‘building’ the 3D virtual models [1, 2, 4–8]. The resolution of acquisition, that is, the minimum difference an instrument is capable of measuring (i.e. sensitivity of the instrument) is also important; however, it depends on the cameras inside the scanner, which are generally very powerful.

To date, the scientific literature considers the accuracy of optical impressions clinically satisfactory and similar to that of conventional impressions in the case of single-tooth restoration and fixed partial prostheses of up to 4–5 elements [18, 19, 21, 24, 35–49]. In fact, the trueness and precision obtained with the optical impressions for these types of short-span restorations are comparable to those obtained with conventional impressions [35–49]. However, optical impressions do not appear to have the same accuracy as conventional impressions in the case of long-span restorations such as partial fixed prostheses with more than 5 elements or full-arch prostheses on natural teeth or implants [6–8, 35–50]. The error generated during intraoral scanning of the entire dental arch does not appear compatible with the fabrication of long-span restorations, for which conventional impressions are still indicated [6–8, 35–49].

However, the latest-generation scanners are characterised by very low errors in full-arch impressions [4], and in this sense the data in the literature must be interpreted critically, as preparing and publishing a scientific article generally takes time, whereas manufacturers release new powerful software for mesh construction very frequently.

#### 3. What are the differences between the optical impression systems available commercially?

To date, only a few studies have compared the trueness and precision of different IOS [4, 50–58]. Almost all are in vitro studies based on models [4, 50–58], as it is currently not possible to calculate the trueness of IOS in vivo; in addition, these studies have quite different experimental designs [50–58]. Some focused on the accuracy of the IOS in dentate models [50, 52, 53, 55–57], while others evaluated the accuracy of the IOS in oral implantology [4, 51, 54, 58]. Regardless, the upshot of these studies is that different IOS have different accuracy; therefore, some devices seem to have more indications for clinical use (for making impressions for fabricating long-span restorations) while others appear to have more limited clinical applications (for making single or short-span restorations) [50–58]. It is very difficult to compare the results (in terms of trueness and precision) of these studies, as scanners have different image-capture technologies and may therefore require different scanning techniques [4, 54, 59, 60]; unfortunately, little is known about the influence of scanning technique on the final results [59–61], and the scientific literature should address this topic in the coming years.

Trueness and precision, however, are not the only elements that can differentiate the devices currently available commercially [1, 2, 4, 7, 34, 54, 59, 62]. A whole series of elements (necessity of opacization with powder, scanning speed, tip size, ability to detect in-colour impressions) differentiate IOS in terms of their clinical use [1, 2, 4, 54, 62]. In particular, scanning systems can differ based on the possibility of whether there is a free interface with all available CAD software (open versus closed systems) and the purchase/management costs [1, 2, 4, 54, 62].

The need for powder and opacization is typical of the first-generation IOS; the more recently introduced devices can detect optical impressions without using powder [2, 4, 34, 62, 63]. Technically, a scanner that allows the clinician to work without opacization should be preferred; in fact, powder may represent an inconvenience for the patient [2, 4, 34, 62, 63]. In addition, applying a uniform layer of powder is complex [2, 34, 62, 63]. An inappropriate opacization technique may result in layers of different thicknesses at various points of the teeth, with the risk of errors that reduce the overall quality of the scan [2, 34, 62, 63].

Scanning speed is certainly a matter of great importance for an IOS [2, 4, 50, 54, 62]. IOS have different scanning speeds, and the latest-generation devices are generally faster than the oldest ones. However, the literature has not clarified which device can be more efficient: in fact, the scanning speed does not depend only on the device, but largely on the experience of the clinician [2, 4, 34, 50, 54, 62].

The size of the tip plays a role as well, especially in the case of second and third molars (i.e. the posterior regions of the maxilla/mandible) [2, 4, 12–18, 34, 62]. A scanner with a tip of limited dimensions would be preferable for the patient’s comfort during the scan; however, even scanners with more voluminous tips allow excellent scanning in posterior areas [2, 4, 12–18, 34, 62].

The possibility of obtaining in-colour 3D models of the dental arches represents one of the latest innovations in the field of optical scanning [1, 2, 4, 28, 34, 64]. To date, only a few IOS can make in-colour impressions. Generally, colour is simply added to the 3D models derived from the scan, overlaying these with high-resolution photographs. The information on colour is meaningful especially in communication with the patient, and is therefore of less clinical importance [1, 2, 4, 28, 34, 64]; in the future, it is possible that IOS will include functions that are now the prerogative of digital colorimeters.

Finally, an IOS should be able to fit in an ‘open’ workflow and should have an affordable purchase and management price [1, 2, 4, 54]. Ideally, an IOS should have two outputs: a proprietary file with legal value, and an open-format file (e.g.. STL,. OBJ,. PLY). Open-format files can be immediately opened and used by all CAD prosthetic systems [1, 2, 4, 54]. In such cases, the literature generally refers to an ‘open system’ [1, 2, 4, 54]. The advantage of these systems is versatility, together with a potential reduction of costs (there is no need to buy specific CAD licenses or to pay to unlock the files); however, a certain degree of experience may be required, initially, to interface the different software and milling machines [1, 2, 4, 54, 62]. This problem does not arise in the case of IOS within a ‘closed system’. Such scanners have as output only the reference proprietary (closed) file, which can be opened and processed only by a CAD software from the same manufacturing company. The inability to freely dispose of. STL files, or the need to pay fees to unlock them, certainly represents the main limits of closed systems [1, 2, 4, 54, 62]. However, the inclusion within an integrated system may encourage workflow, especially in the case of less experienced users. In addition, some closed systems offer a complete, fully integrated digital workflow, from scanning to milling, and provide chair-side solutions. Finally, converting files (e.g. the conversion of proprietary files to open formats) may result in loss of quality and information [2, 62].

The most important features an IOS should have are summarised in Table 2.

**Table 2:** The positive and negative features of commercially available IOS

Feature	Positive	Negative
Trueness	High	Low
Precision	High	Low
Resolution	High	Low
Need for opacization	Powder not needed	Powder needed
Scanning speed	Fast	Slow
Tip	Small/thin	Large/thick
In-colour images	Yes	No
System	Open (free. STL and. PLY files)	Closed (only proprietary files as output) or semi–closed (pay per. STL file)

#### 4. To date, what are the clinical applications of IOS?

IOS are of great utility and are applied in various fields of dentistry, for diagnosis and for fabricating restorations or custom devices in prostheses, surgery and orthodontics [65–132]. IOS are in fact used for acquiring 3D models for diagnostic purposes [2, 4, 6]; these models can be useful for communicating with the patient [2, 6]. Diagnosis and communication are not, however, the only fields of application for IOS. In prostheses, IOS are used to make impressions of preparations of natural teeth [6–8, 65–88] for fabricating a wide range of prosthetic restorations: resin inlays/onlays [65, 66], zirconia copings [67, 68], single crowns in lithium disilicate [69–74], zirconia [19, 75–77], metal-ceramic [78] and all-ceramic [79–81] as well as frameworks and fixed partial dentures [82–87]. Several studies [69–81] and literature reviews [88] have shown that the marginal gap of ceramic single crowns made from intraoral scans is clinically acceptable and similar to that in crowns produced from conventional impressions. The same considerations can be extended to short-span restorations such as fixed partial dentures of three to five elements [36, 82–87], obviously considering the differences stemming from the different accuracies of various IOS. To date, the literature does not support the use of IOS in full-arch impressions: several studies and literature reviews have shown that the accuracy of IOS is not yet sufficient in such challenging clinical cases [7, 8, 35, 37, 39].

In prosthodontics, IOS can be successfully used to capture the 3D position of dental implants and to fabricate implant-supported restorations [4, 14, 17, 18, 21, 24, 47, 51, 54, 58]. The 3D position of the implants captured with the IOS is sent to the CAD software, where the scanbodies are coupled with an implant library, and the desired prosthetic restorations can be drawn within minutes; this restoration then can be physically realised by milling through a powerful CAM machine using ceramic materials [89–119]. At present, implant-supported single crowns [21, 22, 89–104], bridges [104–113] and bars [114–116] can be successfully fabricated from optical impressions. Similar to what the literature has found for natural teeth [6–8, 35, 37], the only apparent limitation to the use of IOS in implant prosthodontics is that of long-span restorations on multiple implants (such as long-span bridges and fixed full arches supported by more than four implants): at least, this is what emerges from the most important reviews [39, 117, 118] and from different in vitro studies on trueness and precision, which indicate that conventional impressions are the best solution for these challenging clinical situations [4, 49, 54, 58].

At present, only a few studies have addressed the use of IOS for fabricating partially [119, 120] and completely [57, 121] removable prostheses; in particular, this last application still presents some issues due to the absence of reference points and the impossibility of registering soft tissue dynamics. However, IOS can be successfully used for digital smile design applications [122], post and core fabrication [123] and for fabricating obturators, in complex cases [124, 125].

Dentogingival model scanning can be superimposed onto files from cone beam computed tomography (CBCT) too, via specific software to create a virtual model of the patient [126–130]. This model is used for planning the positioning of the implants and to draw one or more surgical stents useful for placing the fixtures in a guided manner [126–130]. The use of IOS in this sense has supplanted the old technique of double scanning with CBCT only, which was based on radiologic scans of the patient and of the patients’ plaster models. In fact, the scanning resolution of CBCT is lower than that of IOS; the use of IOS therefore allows the detection of all details of the occlusal surfaces with greater accuracy. This can make the difference in, for example, the preparation of tooth-supported surgical templates. However, care should be taken, as the use of IOS in guided surgery is only in its infancy.

Finally, IOS represent a very useful tool in orthodontics for diagnosis and treatment planning [3, 5, 6, 12, 15, 16, 25, 27, 131, 132]. In fact, optical impressions can be used as a starting point for the realisation of a whole series of customised orthodontic devices, among which aligners should be mentioned [3, 5, 6, 12, 15, 16, 25, 27, 131, 132]. In the coming years, it will be probable that almost all orthodontic appliances will be designed from an intraoral scan, so they will be entirely ‘custom’ and adapted to the patient’s specific clinical needs [3, 5, 6, 12, 15, 16, 25, 27, 131, 132].

The most important clinical indications and contraindications on the use of IOS are summarised in Table 3.

**Table 3:** Clinical indications and contraindications of IOS

Field	Indication	Contraindication
Prosthodontics	Resin inlays/onlays [65, 66]	Long-span fixed partial dentures and/or fixed full arches (6–8 elements) [7, 8, 35, 37, 39]
	Zirconia copings [67, 68]	Long-span implant-supported fixed partial dentures and/or fixed full arches (6–8 implants) [39, 117, 118]
	Single-tooth restorations in lithium disilicate [69–74], zirconia [19, 75–77], all ceramic [79–81]	Complete removable prostheses [57, 121]
	Frameworks and fixed partial dentures in zirconia (4–5 elements) [82–87]	
	Single-implant crowns [21, 22, 89–104]	
	Implant bridges (4–5 implants) [104–113]	
	Implant-supported bars (≤4 implants) [114–116]	
	Posts and cores [123]	
	Partial removable dentures [119, 120]	
	Digital smile design [122]	
	Obturators [124, 125]	
Implantology	Guided implant surgery [126–130]	
Orthodontics	Diagnosis and treatment planning [3, 5, 6, 12, 15, 16, 25, 27, 131, 132]	
	Aligners [3, 132]	
	Custom-made devices [3, 132]	
	The virtual patient [130]	

## Conclusions

Several important elements have emerged from this present narrative literature review, which has examined 132 scientific papers on the topic of IOS and that were published from January 2007 to July 2017.

First, optical impressions have several advantages over conventional impressions: among them, the most important is the reduction of patient stress and discomfort. In fact, many patients today have anxiety and a strong gag reflex and therefore do not tolerate the conventional impressions; in these cases, using light to substitute trays and materials is an ideal solution. Optical impressions, moreover, are time-efficient and can simplify clinical procedures for the dentist, especially for complex impressions (in patients with undercuts and/or in oral implantology, when multiple implants are present). In addition, optical impressions eliminate plaster models, saving time and space, and allow for better communication with the dental technician. Finally, IOS improve communication with patients and are therefore a powerful marketing tool for the modern dental clinic. Conversely, the disadvantages of using optical impressions are the difficulty in detecting deep margin lines in prepared teeth and/or in the case of bleeding, the learning curve, and the purchasing and managing costs.

Regarding accuracy as compared to conventional impressions, optical impressions are equally accurate for individual restorations or 3–4-element bridges on natural teeth and on implants; conversely, conventional impressions still appear to be the best solution currently for long-span restorations, such as fixed full arches on natural teeth and implants (with a higher number of prosthetic abutments).

The IOS currently available commercially differ in terms of accuracy; therefore, the latest-generation devices may have wider indications for clinical use, whereas the oldest have fewer clinical indications. This is an important aspect to be considered before buying an IOS, in addition to other features such as the need for opacization, scanning speed, wand dimensions and possibility of obtaining in-colour images. Technically, the IOS can be integrated in a closed system, generating proprietary files only, or can be open, producing files (.STL,. OBJ,. PLY) that can be opened using any CAD software. In the latter, there will be greater versatility of use, but an integrated proprietary system can undoubtedly be helpful for the less-experienced user.

Finally, the current clinical applications of IOS are extremely wide, as these devices can not only be used in fixed prosthodontics to obtain the virtual models needed to manufacture a whole range of prosthetic restorations (single crowns, fixed partial dentures) on natural teeth and implants, but also in implantology for guided surgery and in orthodontics. At present, the literature does not support using IOS for fabricating long-span restorations, such as fixed full arches supported by natural teeth or implants. In the near future, the dentogingival information captured with IOS will be added to the bone tissue information obtained by CBCT. Along with the information of the patient’s face captured with a face scanner, this will allow clinicians to integrate different file formats into a single model that can be used for surgical, prosthetic and orthodontic planning: this will be the ‘virtual patient’.

The present study has its limitations, as it is only a narrative review, and more systematic reviews of the literature are certainly needed to draw more specific conclusions about the accuracy and clinical indications of IOS in prosthetic and implant dentistry as well as in orthodontics. Further randomised controlled studies on the use of IOS are needed to be able to perform a systematic analysis of the literature that can rely on an adequate number of cases/patients treated effectively.

## Abbreviations

3D: Three-dimensional
CAD: Computer-assisted-design
CAM: Computer-assisted-manufacturing
CBCT: Cone Beam Computed Tomography
DDS: Digital Dentistry Society
IOS: Intraoral Scanners
OBJ: Object File or 3D Model Format
PLY: Polygon File Format or Stanford Triangle Format
STL: Standard Tessellation or Stereolithographic File
